# Recent advances in epigenetic anticancer therapeutics and future perspectives

**DOI:** 10.3389/fgene.2022.1085391

**Published:** 2023-01-04

**Authors:** Liwen Ren, Yihui Yang, Wan Li, Hong Yang, Yizhi Zhang, Binbin Ge, Sen Zhang, Guanhua Du, Jinhua Wang

**Affiliations:** ^1^ The State Key Laboratory of Bioactive Substance and Function of Natural Medicines, Beijing, China; ^2^ Key Laboratory of Drug Target Research and Drug Screen, Institute of Materia Medica, Chinese Academy of Medical Science and Peking Union Medical College, Beijing, China

**Keywords:** epigenetics, cancer, histone, epigenetic drug, combined pharmacotherapy

## Abstract

Tumor development is frequently accompanied by abnormal expression of multiple genomic genes, which can be broadly viewed as decreased expression of tumor suppressor genes and upregulated expression of oncogenes. In this process, epigenetic regulation plays an essential role in the regulation of gene expression without alteration of DNA or RNA sequence, including DNA methylation, RNA methylation, histone modifications and non-coding RNAs. Therefore, drugs developed for the above epigenetic modulation have entered clinical use or preclinical and clinical research stages, contributing to the development of antitumor drugs greatly. Despite the efficacy of epigenetic drugs in hematologic caners, their therapeutic effects in solid tumors have been less favorable. A growing body of research suggests that epigenetic drugs can be applied in combination with other therapies to increase efficacy and overcome tumor resistance. In this review, the progress of epigenetics in tumor progression and oncology drug development is systematically summarized, as well as its synergy with other oncology therapies. The future directions of epigenetic drug development are described in detail.

## 1 Introduction

Cancers are the second leading cause of human death, second only to cardiovascular disease (Siegel et al., 2021). The origination and development of cancer is usually a synergistic effect of epigenetic alterations, genetic mutations, accompanied with environmental factors. Epigenetic regulation is distinguished from genetic mutation and refers to a form of regulation that can regulate gene expression without alteration of DNA sequence (Bird, 2007). Epitranscriptomics has emerged as another level of epigenetic regulation similar to DNA and histone modifications. The epitranscriptomic regulation refers to the relevant functional changes of the transcriptome without any alteration of the RNA sequence (Meyer et al., 2012). Recent studies have found that epigenetic regulation and relevant therapeutics play an irreplaceable role in the mechanism research of cancer occurrence and development and in the process of cancer treatment.

Due to the heterogeneity of tumor cells, tumor recurrence and drug resistance frequently occur, which are the main reasons for the high mortality of cancer. In the early stage of tumor development, numbers of epigenetic changes occur in tumor cells (Ka-Yue Chow et al., 2022; Zandieh et al., 2022). Therefore, it is essential to find drugs that can regulate the abnormal epigenetic regulation of tumor cells. Epigenetic regulation of genes includes DNA methylation, RNA methylation, histone modifications and non-coding RNAs. At present, great progress has been made in the development of antitumor drugs targeting various epigenetic regulation, and multiple drugs have entered the clinical use or clinical research stage.

Although epigenetic drugs have made great progress in the treatment of hematological tumors, they are less effective in solid tumors. With the proposal of drug combination regimens, the combination of epigenetic drugs and other therapies has achieved good efficacy in several solid tumors, such as radiation therapy, chemotherapy, hormone therapy, targeted therapy and immunotherapy. Numerous completed and ongoing clinical trials have been conducted to evaluate the plausibility of combination schemes integrating epigenetic drugs.

It is of great significance to analyze the mechanism of gene irregulation in cancer cells and to identify agents that could modify the abnormal expression of genes. In this review, we reviewed the recent progress of epigenetics in tumor progression and anticancer therapeutics development. In addition, the combination of epigenetic drugs and other oncology therapies are specially reviewed.

## 2 Epigenetic phenomenon and cancer

### 2.1 DNA methylation and cancer

DNA methylation is the firstly recognized epigenetic alterations and it is closely connected with the development of cancer. When the promoter region of genes was methylated, the accessibility to regulatory regions in the DNA was blocked and the transcription factors or other transcriptional regulators can't bind with the promoter of genes, which lead to the repression of gene transcription (Jurmeister et al., 2022). Specifically, various tumor suppressor genes (TSGs) were identified to be hypermethylated thus facilitating the development of cancer *via* TSGs silencing, such as BRCA1 (Das et al., 2022) and CDKN2A (Maeda et al., 2003). On the contrary, hypomethylation of the DNA will lead to the overexpression of genes. It will turn on the expression of oncogenes which contributes to the tumorigenesis (Beetch et al., 2021). Furthermore, the abnormal DNA methylation, such as site-specific hypermethylation and genome-wide hypomethylation, are frequently recognized in the CpG islands of the gene regulatory region of tumor cells (Saghafinia et al., 2018).

The process of DNA methylation is modulated by the DNA methyltransferases (DNMTs) family, which contains DNMT1, DNMT2, DNMT3A, DNMT3B and DNMT3L (Tajima et al., 2016). DNMT1 is the most plentiful enzyme in the DNMTs family which accounts for modulating the methylation of newly synthesized DNA (Lee et al., 2001), while the DNMT3 enzymes primarily participates in *de novo* methylation (Chedin, 2011). Abnormal alterations of 5-methylcytosine (5 mC) could indorse unrestrained cell propagation thus promoting tumor progression. The ten-eleven translocation (TET) family of DNA hydroxylases could catalyze 5 mC to various oxidative mediates, such as 5-formylcytosine (5 fC), 5-hydroxymethylcytosine (5hmC) and 5-carboxylcytosine (5caC) and unmethylated cytosine (Strzyz, 2022). The related enzymes of methylation are recognized as potential targets of cancer.

### 2.2 RNA modifications and cancer

The methylation in the N6-position of adenosine on eucaryotic mRNA (N6-methyladenosine, m6A) could regulate the metabolism of RNA, such as splice, transport, degradation, translation and miRNA modulation (Wang et al., 2020a). Recent studies suggested that m6A could modulate the proliferation, apoptosis and metastasis of cancer cell, through regulating the cancer-associated genes (He et al., 2019). There are three main types of regulators responsible for m6A regulation, including writers, readers and erasers. The methyltransferase complex (MTC) is the writer to catalyze the methylation of mRNA, whereas the demethylase erases the m6A. The MTC takes charge of the catalysis of m6A, which include METTL3 and other assistant units (Jansens et al., 2022). And the RNA reader protein identified the m6A to exert relevant effects (Zhou et al., 2020). The eraser is demethylase which eliminates m6A with α-ketoglutarate as co-substrate and ferrous iron as cofactor. FTO and ALKBH5 are the identified m6A erasers so far. FTO could regulate the splicing of mRNA *via* blocking the binding of SRSF2 at RNA splice sites (Zhao et al., 2014). Many FTO inhibitors have been found to have antitumor effects and are currently in preclinical studies.

Multiple evidence suggested that the m6A modification has dual role in cancer. The m6A modification promote tumor progression *via* upregulating the expression of oncogenes or inhibiting the expression of tumor suppressor genes. On the contrary, the m6A modification could also inhibit the expression of oncogenes and elevate the expression of tumor suppressor genes (He et al., 2019).

### 2.3 Histone modifications and cancer

Histone Modifications could regulate the accessibility and conformation of chromatin thereby modulating gene expression (Morgan and Shilatifard, 2020). The positively charged histone proteins offer competent integration with DNA of negative charge. The N-terminal of histone proteins are abundant in arginine and lysine residues that could be frequently modified (Zhou et al., 2019). The related histone-modifying enzymes modify the relevant residues of the tails of histone *via* methylation, acetylation, phosphorylation. Besides, histone modifications are being discovered gradually, such as the ubiquitination, citrullination, ADP-ribosylation, formylation, deamination, propionylation, O-GlcNAcylation, butyrylation, proline isomerization, crotonylation and lactylation. There are three types of proteins interacted with the histone, (I) the readers which identify the modifications of histone, (II) the writers which regulate the modifications of histone, (III) the erasers which remove the modifications of histone (Millan-Zambrano et al., 2022).

#### 2.3.1 Acetylation of histone

Histone acetylation is modulated *via* histone deacetylases (HDACs) and histone acetyltransferases (HATs) in a reversible and dynamic way (Icardi et al., 2012). The primary function of HATs is adding the acetyl group (-CH_3_CO) to lysine residues which are related to the activation of gene transcription. On the contrary, the HDACs are erasers which are responsible for removal of the acetyl groups (Shvedunova and Akhtar, 2022).

The charge neutralization model was applied for the explanation of the mechanism of histone acetylation. Histones tightly bind with negatively charged DNA through the lysine residues with positive charge on H3/H4. When histones are acetylated, chromatin configuration will no longer be tight and transformed to euchromatin with loose state. Therefore, the transcriptional factors (TFs) will be recruited for activation of gene transcription (Nicolas et al., 2018). In contrast, HDACs could remove the acetylation of lysine residues and the configuration of chromatin converts to condensed heterochromatin. The acetylation of H4 at the lysine-16 (H4K16) is essential for the chromatin folding and the transition of euchromatin to heterochromatin (Wang et al., 2020b). In addition, the acetylation of histone could provide the binding site for the proteins which participate in the activation of genes, such as the proteins of the bromodomain-containing family (Qin et al., 2019).

Bromodomain and extraterminal domain (BET) proteins are readers of the acetylated proteins, which contains a couple of tandem bromodomains, a C-terminal domain and an extra-terminal domain. The BRD family includes BRD2, BRD3, BRD4 and BRDT. The first three are commonly distributed in tissues, and BRDT is only expressed in the testis (Boyson et al., 2021). The BET families are principally responsible for the recognition of the acetylation of histone H4, but also recognize the acetylation of non-histone proteins, like transcription factors. For example, BRD4 could bind with the TWIST which is an essential transcription factor in the metastasis of cancer (Shi et al., 2014). It also plays an important part in the regulation of oncogene MYC (Devaiah et al., 2020). The inhibitors of BET (BETi) are recognized as an important item for the research and development of antitumor drugs.

#### 2.3.2 Methylation of histone

Histones can be methylated at the arginine or lysine residues which are mediated *via* the histone methyl transferases (HMTs), whereas the histone demethylases (HDMs) regulate the elimination of methylation. The consequence of histone methylation can be repression or activation of transcription, depending on the methylated residues (Black et al., 2012). In general, trimethylation of lysine 4 on H3 (H3K4me3) (Hughes et al., 2020) signifies activation of gene transcription, whereas the trimethylation of lysine 9 (H3K9me3) (Feng et al., 2020) and 27 (H3K27me3) (Raas et al., 2022) on H3 represents inhibition of gene transcription. EZH2 belongs to the polycomb repressive complex 2 (PRC2), which is responsible for the catalysis of methylation of lysine 27 of histone H3 (Pan et al., 2016; Jiang et al., 2021). EZH2 is an essential therapeutic target of various cancers, and multiple inhibitors of EZH2 have entered clinical or preclinical studies. Furthermore, the levels of lysine methylation are also related to the transcription repression or activation, which could be identified *via* diverse methyl-lysine-binding domains. Tumor cells are usually found to possess abnormal histone modifications at single gene or global nuclei levels (Cornett et al., 2019).

## 2.4 Non-coding RNAs

The sequencing of the entire human genome has shown that only ∼2% of the genome is translated. The non-coding RNAs (ncRNAs) could be generally characterized to small and large ncRNAs (lncRNA, more than 200 nucleotides) (Anastasiadou et al., 2018). These ncRNAs were identified as an essential regulator in the development of various disease including cancer (Esteller, 2011). The small ncRNAs comprise small interfering RNAs (siRNAs), PIWI interacting RNAs (piRNAs), microRNAs (miRNAs) and small nucleolar RNAs (snoRNAs). The small ncRNAs are participated in the silencing of targeted gene with high level of sequence conservation among different species (Matsui and Corey, 2017). On the contrary, the lncRNAs possess low level of sequence conservation across species and the mechanisms in the transcription regulation are more complicated (Zhu et al., 2013). Particularly, the lncRNAs is identified as molecular scaffolds for the multiple regulators of chromatin (Rinn, 2014), whereas the function is disrupted in the various cancers. The lncRNA HOTAIR was found to be upregulated in multiple cancers (Qu et al., 2019) and act as a molecular scaffold for the PRC2 complex to target the chromatin (Tsai et al., 2010). Silencing of HOTAIR could inhibit the metastasis of colorectal cancer and breast cancer *via* regulating PRC2 occupancy (Kogo et al., 2011).

## 3 Epigenetic therapeutics for cancer

### 3.1 DNA methyltransferase inhibitor

The DNMT inhibitors are classified to two types generally: nucleoside analogues and non-nucleoside analogues. The nucleoside analogues are modified molecule of cytidine which could covalently interact with the catalytic positions of DNMTs in an irreversible way (Yu et al., 2019). Two DNA methyltransferase inhibitors (DNMTi), 5-azacitidine (Vidaza) and its deoxyanalogue decitabine (Dacogen), have been approved for clinical use, which increases survival time and ameliorates life quality of patients. Azacitidine (Cogle et al., 2015) and decitabine (Dhillon, 2020) are usually used for the treatment of myelodysplastic syndrome (MDS), acute myeloid leukemia (AML) or chronic myelomonocytic leukemia (CMML). The derivate of decitabine, SGI-110, is a novel hypomethylating compound for the treatment of AML and MDS that has undergone phase II clinical trial (Garcia-Manero et al., 2019a). CP-4200 was designed as a pro-drug of azacytidine. It was an elaidic acid ester for azacytidine, which exerted better therapeutic effect than azacytidine (Brueckner et al., 2010). Besides, RX-3117 was also a nucleoside analogue which could suppress DNMT1 and could inhibit the proliferation of cancer *in vivo* (Balboni et al., 2019). Unfortunately, overall hypomethylation of genome could happen due to the non-specificity of nucleoside analogues (Flausino et al., 2021). Therefore, some non-nucleoside inhibitors of DNMTs are exploited. The non-nucleoside inhibitors can bind the catalytic site of DNMTs without binding the DNA directly. Hydralazine which is indicated for the management of hypertension has been studied for its potential as a DNMT inhibitor. It was demonstrated that in prostate cancer cells hydralazine treatment lowered the production of DNMT1, DNMT3a and DNMT3b mRNA suggesting its potential in reducing the malignant growth through epigenetic alteration (Graca et al., 2014). An antisense oligonucleotide designed to bind with the 3′ untranslated region of DNMT1 mRNA and hindering with its transcription is MG98. It is a second generation DNMT inhibitor specifically inhibiting DNMT1 without altering DNMT3 expression. Clinical study has been carried out with MG98 in combination with interferon for the treatment of metastatic renal cell carcinoma and was proven to be safe at a particular dosage (Amato et al., 2012). SGI-1027 is a derivative of quinoline which could suppress DNMT1, DNMT3A and DNMT3B without binding with DNA. SGI-1027 could upregulate the TSGs of which the transcription is blocked in tumor cells (Sun et al., 2018) (Table 1).

**TABLE 1:** Epigenetic anticancer therapeutics.

Type	Status	Category	Compound	Applications	Reference
DNMTi	Approved	Nucleoside analogues	5-azacitidine	MDS, AML,CMML	Cogle et al. (2015)
	Approved	Nucleoside analogues	Decitabine	MDS, AML,CMML	Dhillon, (2020)
	Phase II	Nucleoside analogues	SGI-110	MDS, AML	Garcia-Manero et al. (2019a)
	Phase I/II	Nucleoside analogues	RX-3117	Pancreatic cancer	Balboni et al. (2019)
	Preclinical (*In vitro*, *In vivo*)	Nucleoside analogues	CP-4200	AML	Brueckner et al. (2010)
	Phase I/II	Non-nucleoside analogues	Hydralazine	MDS, CTCL, solid tumors	Graca et al. (2014)
	Phase I/II	Non-nucleoside analogues	MG98	DMS, AML, renal cancer	Amato et al. (2012)
	Preclinical (*In vitro*)	Non-nucleoside analogues	SGI-1027	Solid tumors	Sun et al. (2018)
HMTi	Phase I	DOT1L inhibitor	EPZ-5676	Hematological malignancy	Gao and Ge, (2018)
	Preclinical (*In vitro*)	DOT1L inhibitor	EPZ004777	Mixed lineage leukemia	Liu and Wang, (2016)
	Phase II	EZH2 inhibitor	Tazemetostat	Lymphoma and solid tumors	Duan et al. (2020)
	Preclinical (*In vitro*, *In vivo*)	EZH2 inhibitor	EPZ005687	Lymphoma	Girard et al. (2014)
	Preclinical (*In vitro*)	EZH2 inhibitor	DZNeP	Colon, breast cancer	Zauderer et al. (2022)
	Preclinical (*In vitro*)	SMYD2 inhibitor	AZ505	Glioma	Kojima et al. (2020)
	Preclinical (*In vitro*)	SMYD2 inhibitor	LLY-507	Ovarian clear cell carcinoma	Knutson et al. (2014)
	Preclinical (*In vitro*)	SMYD2 inhibitor	A-893	Lung cancer	Pan et al. (2022)
	Preclinical (*In vitro*, *In vivo*)	G9a inhibitor	BIX-01294	Colon cancer	Padeken et al. (2022)
	Preclinical (*In vitro*, *In vivo*)	G9a inhibitor	UNC0638	Renal cancer	Chae et al. (2019)
HDMi	Phase I	LSD1 inhibitor	Tranylcypromine analogue	AML	Ojha et al., (2021); Wass et al., (2021)
	Preclinical (*In vitro*, *In vivo*)	LSD1 inhibitor	Pargyline	Prostate cancer	Fang et al. (2019)
	Preclinical (*In vitro*)	LSD1 inhibitor	Polyamine analogues	Breast cancer	Dai et al. (2020)
	Preclinical (*In vitro*, *In vivo*)	LSD1 inhibitor	Namoline	Prostate cancer	Sharma et al. (2010)
	Preclinical (*In vitro*, *In vivo*)	LSD1 inhibitor	HCI-2509	Prostate cancer	Sharma et al. (2010)
HATi	Preclinical (*In vitro*, *In vivo*)	p300 inhibitor	C646	AML	Li et al. (2022)
	Preclinical (*In vitro*)	Tip60 inhibitor	Anacardic acid	Breast cancer	Ghizzoni et al. (2009)
	Preclinical (*In vitro*, *In vivo*)	Tip60 inhibitor	6-alkyl salicylates	Cancer	Wu et al. (2009)
	Preclinical (*In vitro*, *In vivo*)	Pyridoisothiazole derivative	PU139	Neuroblastoma	Ghizzoni et al. (2012)
	Preclinical (*In vitro*, *In vivo*)	Pyridoisothiazole derivative	PU141	Neuroblastoma	Ghizzoni et al. (2012)
HDACi	Approved	Hydroxamic acid derivatives	Vorinostat	CTCL	Willmann et al. (2012)
	Approved	Hydroxamic acid derivatives	Pracinostat	AML	Bird et al. (2020)
	Approved	Hydroxamic acid derivatives	Panobinostat	Multiple myeloma	Ho et al. (2020)
	Phase I/II	Hydroxamic acid derivatives	Abexinostat	Relapsed/Refractory lymphoma	Garcia-Manero et al. (2019b)
	Phase II	Hydroxamic acid derivatives	Resminostat	Pancreatic cancer, lymphoma	Galli et al. (2010)
	Phase II	Hydroxamic acid derivatives	Givinostat	Multiple myeloma	Ribrag et al. (2017)
	Phase II	Cyclic Peptides	Romidepsin	CTCL, PTCL	Damaraju et al. (2012)
	Phase II	Benzamide derivative	Mocetinostat	MDS, Relapsed/Refractory lymphoma	Witta et al. (2012)
	Phase III	Benzamide derivative	Entinostat	Melanoma, leukemia, breast cancer	(Sborov et al., (2017); Connolly et al., (2021); Ny et al., (2021))
	Preclinical (*In vitro*)	Short chain fatty acids	Phenylbutyrate	Glioblastoma, CRC	Arvidsson et al. (2016)
	Preclinical (*In vitro*)	Short chain fatty acids	Valproic acid	Solid tumors, glioma	Richards et al. (2006)
BETi	Phase I/II	Thienotriazolodiazepine	OTX015	Leukemia	Delmore et al. (2011)
	Phase I/II	Benzoisoxazoloazepine	CPI-0610	Relapsed/Refractory lymphoma	Berthon et al. (2016)
	Preclinical (*In vitro*, *In vivo*)	Benzodiazepene derivative	JQ1	Multiple myeloma	Byun et al. (2019)
m6Ai	Phase I	METTL3 inhibitor	STC-15	Advanced malignancies	Holz, (2022)
	Preclinical (*In vitro*, *In vivo*)	METTL3 inhibitor	STM2457	AML	Yankova et al. (2021)
	Preclinical (*In vitro*)	FTO inhibitor	MA	Cervical cancer	Huang et al. (2015)
	Preclinical (*In vitro*, *In vivo*)	FTO inhibitor	MA2	Glioblastoma	Xiao et al. (2020)
	Preclinical (*In vitro*)	FTO inhibitor	FB23-2	AML	Huang et al. (2019)
	Preclinical (*In vitro*)	FTO inhibitor	R-2HG	Leukemia, glioma	Dang et al. (2009)

### 3.2 RNA methyltransferase inhibitor

Studies suggested that inhibition of m6A was able to facilitate development of various cancers. So far, the first METTL3 inhibitor, STC-15, has entered phase I clinical trials for the treatment of advanced malignancies (Holz, 2022). STM2457, which is also an inhibitor of METTL3, leads to reduced AML growth, and an increase in differentiation and apoptosis of AML cells *in vitro*. Furthermore, STM2457 could also contribute to impaired engraftment and prolonged survival in various AML mouse models (Yankova et al., 2021). Meclofenamic acid (MA) is a selective inhibitor of FTO *via* preempting binding sites of FTO (Huang et al., 2015). MA2 is an ethyl ester derivative of MA and it could inhibit the proliferation of glioblastoma stem-like cell both *in vitro* and *in vivo* (Xiao et al., 2020). FB23-2 was also identified as an inhibitor of FTO. It could promote the differentiation and inhibit the proliferation of AML cells (Huang et al., 2019). R-2- hydroxyglutarate (R-2HG) is a metabolite of mutant IDH1/2 enzymes, which increased the m6A level and accelerated the degradation of oncogenes (Dang et al., 2009). The research of m6A is an emerging field. Currently, the research and development of m6A inhibitors are in the pre-clinical stage. It is believed that many m6A inhibitors will enter the clinical trials or even market stage in the future (Table 1).

### 3.3 Drugs regulating histone modification

#### 3.3.1 Inhibitors of histone methyltransferases

HMTs are identified to be highly expressed in a variety of cancers, indicating HMTs to become latent therapeutic target for cancers (Liu and Wang, 2016). The inhibitor of lysine methyltransferase DOT1L (Vatapalli et al., 2020), EPZ004777, was designed basing on the S-adenosyl methionine binding domain. It could suppress the activity of DOT1L enzyme, thus downregulating the methylation level at H3K79 (Gao and Ge, 2018). Besides, EPZ-5676 was also a DOT1L inhibitor which could significantly inhibit the progression of leukemia *via* reducing the methylation of H3K27 (Waters et al., 2015). EZH2 is the main element of PRC2 which is related to the H3K27 methylation, contributing to the inhibition of TSGs. EZH2 was found to upregulate in various cancers, such as breast cancer and prostate cancer (Duan et al., 2020). EZH2 inhibitor tazemetostat have been proven effective in patients with relapsed or refractory, BAP1-inactivated malignant pleural mesothelioma in a multicentre, open-label, phase 2 study (Zauderer et al., 2022). The S-adenosyl-L-homocysteine hydrolase inhibitor DZNep could degrade the expression of EZH2 and inhibit the proliferation and metastasis of chondrosarcoma (Girard et al., 2014). EPZ005687 and EPZ-6438 (Knutson et al., 2014) are selective inhibitors of EZH2 which possess excellent inhibitory activity against lymphoma. SMYD2 is another lysine methyltransferase which mainly modulate the methylation of H2B, H3 and H4. Several inhibitors of SMYD2, like LLY-507 (Kojima et al., 2020), AZ505 (Pan et al., 2022) and A-893 (Sweis et al., 2015) could significantly suppress the proliferation of various cancer cells. The methyltransferase G9a is responsible for the methylation of H3K9 (Padeken et al., 2022). It is overexpressed in various cancers and its inhibitors, BIX-01294 (Chae et al., 2019) and UNC0638 (Li et al., 2021) are able to inhibit the activity of G9a selectively with anti-tumor effects. Studies have found that Set 7/9 could both regulate the methylation of H3K4 and estrogen receptor (ER). Cyproheptadine was recognized as a Set 7/9 inhibitor which could inhibit the proliferation of breast cancer cells by modulating the expression of ER (Takemoto et al., 2016) (Table 1).

#### 3.3.2 Inhibitors of histone demethylase

There are two main categories of the inhibitors of HDMs. One type is the Lysine-specific demethylases LSD1/2 with the amine oxidases properties, belonging to the HDM1 subgroup. The remaining HDM2-8 subgroups contains jumonji C domain which is α-ketoglutarate and iron dependent (Nowak et al., 2016).

A variety of LSD1 inhibitors are currently in clinical or preclinical studies (Fang et al., 2019). LSD1 inhibitor pargyline was reported to suppress the growth and epithelial-to - mesenchymal transformation (EMT) of prostate carcinoma cells (Ojha et al., 2021). The antidepressant drug tranylcypromine was also identified as a LSD1 inhibitor with antineoplastic activity (Wass et al., 2021). Besides, there are several LSD1 inhibitors derivated on tranylcypromine structure undergoing clinical research for the treatment of Leukemia (Dai et al., 2020). The derivatives of polyamine could upregulate the methylation of H3K4 in triple negative breast cancer cells *via* inhibiting LSD1 (Zhu et al., 2012). Similarly, derivatives of biguanides or guanidines could also suppress the activity of LSD1, thus inhibiting the proliferation of lung cancer cells through upregulating H3K4 methylation (Sharma et al., 2010). In addition, LSD1 inhibitors Namolineand HCI-2509 can inhibit the proliferation of prostate cancer in like manner (Willmann et al., 2012).

On the other hand, the derivative of hydroxamic acid SAHA (vorinostat) was proved as an effective inhibitor of KDM4E and its derivative IOX1 was also demonstrated to inhibit various types of HDMs (Siegel et al., 2009). In particular, various flavonoid compounds, such as caffeic acid and myricetin, have presented inhibitory activity on numerous jumonji C HDMs (Li et al., 2022) (Table 1).

#### 3.3.3 Inhibitors of histone acetyltransferase

The HATs play an essential role in the modulation of transcription and are promising therapeutic target of cancer. The HATs inhibitor C646, which could competitively inhibit the activity of p300, could significantly block cell cycle and induce cell apoptosis of acute myeloid leukemia (AML) cells (Gao et al., 2013). The isothiazolone is both the inhibitor of p300 and PCAF and was demonstrated effectively in inhibiting colorectal cancer (Ghizzoni et al., 2009). The natural product anacardic acid was demonstrated as the inhibitor of MYST family (Wu et al., 2009) and its analogs 6-alkylsalicylate was identified as the inhibitor of Tip60 (Ghizzoni et al., 2012). They have been found to inhibit the growth of pancreatic cancer, breast cancer and prostate cancer. Especially, PU139 and PU141, which are derivatives of pyridoisothiazolone, could suppress the activity of p300, CBP, Gcn5 and PCAF both *in vitro* and *in vivo* (Ramakrishnan et al., 2022). The antineoplastic activity of the above compounds was also proved both in neuroblastoma cells and xenografts models in mice (Table 1).

#### 3.3.4 Inhibitors of histone deacetylase

The application of inhibitors of HDAC (HDACis) was successfully proved in the treatment of cancer in clinical practice. The HDACis are able to inhibit the proliferation of cancer cells through inducing cell apoptosis and suppressing the process of EMT by inhibiting the expression related to the cell migration and angiogenesis (Ho et al., 2020).

The derivative of hydroxamic acid Vorinostat was the first HDACi authorized by the Food and Drug Administration (FDA) for the therapeutic of cutaneous T cell lymphoma (CTCL) (Siegel et al., 2009). Since then, numerous derivatives of hydroxamic acid have been developed for preclinical or clinical studies, such as Pracinostat, Abexinostat, Givinosta, Resminostat and Panobinostat (Bird et al., 2020). Pracinostat has been approved for the treatment of AML as a breakthrough therapy, combined with azacytidine (Garcia-Manero et al., 2019b). Besides, Abexinostat has also been approved for the treatment of follicular lymphoma after achieving favorable treatment results in clinical trials (Ribrag et al., 2017). Givinostat has been undergoing phase II clinical trial for the treatment of multiple myeloma (Galli et al., 2010). Similarly, Resminostat has been evaluated for the treatment of relapsed Hodgkin lymphoma in phase II clinical study now (Walewski et al., 2019). In addition, 4SC-202 was a novel HDACi and undergoing in the phase I clinical trial for the treatment of advanced hematological cancers (von Tresckow et al., 2019). Tasquinimod, which is an anti-angiogenic compound for the therapeutic of castration resistant prostate cancer, is identified as an allosteric regulator of HDAC4 (Isaacs et al., 2013). AR-42 is a pan-HDAC inhibitor which is effectively demonstrated in phase I research for the treatment of B-, T-cell lymphomas and multiple myeloma (Sborov et al., 2017).

Another major class of HDACi is the derivatives of benzamide, such as entinostat, mocetinostat and tacedinaline (CI-994). Entinostat was effectively examined without severe toxic effects in breast cancer (Connolly et al., 2021), melanoma (Ny et al., 2021) and metastatic non-small cell lung cancer (NSCLC) (Witta et al., 2012) in phase II/III clinical trials, either alone or in combination with other drugs. Mocetinostat is also in the phase II clinical study which is applied for the metastatic leiomyosarcoma (Choy et al., 2018) and relapsed classical Hodgkin’s lymphoma (Younes et al., 2011) with promising activity with manageable toxicity as single agent. Tacedinaline could inhibit the proliferation of cell lines of NSCLC *in vitro* and tacedinaline will exert better effect when it with combined with other anticancer agents, like docetaxel and gemcitabine (Loprevite et al., 2005). However, results of a phase II multicenter study suggested that gemcitabine combined with tacedinaline presented no benefit than gemcitabine alone in advanced pancreatic cancer patients with advanced pancreatic cancer (Richards et al., 2006).

The valproic acid (VPA) and phenylbutyrate, which belongs to the short chain fatty acid type, are also found to inhibit the activity of HDAC with anti-cancer activity. VPA was effectively demonstrated in the neuroendocrine tumors (Arvidsson et al., 2016). *In vitro* studies have suggested that phenylbutyrate is able to inhibit the proliferation of glioblastomas cells (Ye et al., 2019). Various natural products were found to exert HDAC suppressing activity, such as cyclopeptide, amamistatin and chlamydocin (Byun et al., 2019) (Table 1).

### 3.4 Drugs regulating BET proteins

There are numerous BETis undergoing the clinical or preclinical studies currently. JQ1 is the first designed BETi which could bind with the bromodomains or the acetyl-lysine competitively (Filippakopoulos et al., 2010). JQ1 could arrest cell cycle and induce cell senescence in multiple myeloma through inhibiting the expression of c-Myc (Delmore et al., 2011). Thienotriazolodiazepine OTX015 is the first BETi which enters clinical trials. OTX015 could arrest cell cycle, induce apoptosis of cell and inhibit the growth of acute leukemia cell lines by downregulating BRD2, BRD4 and MYC expression (Coude et al., 2015). In the clinical trials, OTX015 presented favorable therapeutic effects within the tolerable dose in the treatment of AML (Berthon et al., 2016). CPI-0610 is also a BETi with benzoisoxazoloazepine structure which is undergoing phase I clinical trial for the treatment of refractory or relapsed lymphomas (Albrecht et al., 2016) (Table 1).

## 4 Epigenetic drugs in combination with other therapies

### 4.1 Epigenetic drugs in combination with radiotherapy

The combinations of radiotherapy and inhibitors of DNMT, HDAC, BET and EZH2 have been demonstrated to increase the sensitivity of radiotherapy to patients in preclinical research through arresting cell cycle, upregulating oxidative stress and preventing DNA-damage repair. The above studies suggest the great potential of epigenetic drugs in combination with radiotherapy. In a phase I trials, the combination of vorinostat and radiotherapy with capecitabin significantly increased the overall survival of patients of pancreatic ductal adenocarcinoma (Tinari et al., 2012). In addition, the combination of vorinostat and radiotherapy could improve the objective response rate of refractory neuroblastoma (Mueller et al., 2011) and gastrointestinal carcinoma (Ree et al., 2010), compared with using the radiotherapy alone. Unfortunately, not all the epigenetic drugs in combination with radiotherapy will exert the above effect, whereas leading to severe toxic effect sometimes.

### 4.2 Epigenetic drugs in combination with chemotherapy

In preclinical research, the combinations of chemotherapy with the DNMT (Gravina et al., 2010) and HDAC inhibitors (Arrighetti et al., 2015) significantly strengthen the killing effect of chemotherapeutics on tumor cells by promoting DNA damage and inhibiting the repair of DNA damage. Besides, the drug resistance of chemotherapeutics could be overcome when in combinations of DNMT or HDAC inhibitors (Wang et al., 2020c). Unfortunately, although the preclinical experiments suggested that the combination of chemotherapy and epigenetic drugs could improve the efficacy of chemotherapy, the clinical trials frequently presented unfavourable results due to no significant improvement in efficacy accompanied by serious adverse effects (Choy et al., 2015).

### 4.3 Epigenetic drugs in combination with hormone therapy

In the preclinical studies, the HDACi could exaggerate the therapeutic effect and overcome drug resistance of the hormone therapy in breast cancer animal models (Bijian et al., 2018). Besides, the inhibitors of BET could be used in combination with fulvestrant to inhibit the proliferation of tamoxifen-resistant breast cancer cells both *in vitro* and *in vivo* (Li et al., 2020). CPI-1 is a specific inhibitor of CBP and p300 which could combine with the anti-oestrogen therapies for the treatment of breast cancer by inhibiting the ERα pathway (Waddell et al., 2021).

Not only that, the combination of epigenetic drugs and hormone therapy is also proven safely and effectively in the clinical trials. A phase II trial suggested that the patients with endocrine-resistant metastatic breast cancer that treated tamoxifen combined with vorinostat had high therapeutic responses with favorable tolerability (Peterson et al., 2021). Similarly, exemestane combined with entinostat (HDAC inhibitor) significantly improved the progression free survival (PFS) of hormone receptor-positive, advanced-stage, endocrine -resistant breast cancer in postmenopausal women, which was approved by the FDA as a breakthrough therapy (Connolly et al., 2021).

The combination of BET inhibitor JQ1 and anti-androgen enzalutamide could significantly inhibit the proliferation of prostate cancer xenografts which is enzalutamide-resistant (Asangani et al., 2014). Similar results were obtained for the combination of BET inhibitor OTX-015 and AR-agonist ARN-509 (Asangani et al., 2016). At present, these drug combinations are in clinical trial studies. In addition, the addition of HDAC inhibitor (Panobinostat) could overcome the resistance of the second-line anti-androgen therapy of prostate cancer, which remarkably improve the PFS of patients (Ferrari et al., 2019).

### 4.4 Epigenetic drugs in combination with targeted therapy

Preclinical studies suggested that the application of epigenetic drugs could overcome the drug resistance to the HER family receptor tyrosine kinases (RTKs). Using BETs remarkably upregulated the sensitivity of head and neck squamous cell cancer (HNSCC) to anti-EGFR antibody (Leonard et al., 2018) and the sensitivity of HER2-positive breast carcinoma to lapatinib (Stuhlmiller et al., 2015). Unfortunately, the combination of RTK inhibitors and epigenetic drugs usually exhibited greater toxicity in the clinical experiments, which made it difficult to achieve the desired efficacy (Pili et al., 2017).

In addition, numerous clinical trials have demonstrated that the combination of anti-angiogenic therapeutics and HDAC inhibitors could remarkably improve the efficacy of the treatment for various cancers with favorable safety profile. The drug combinations have achieved good efficacy and safety, such as sorafenib and resminostat (HDACi) in treatment of hepatocellular cancer (Bitzer et al., 2016), bevacizumab and vorinostat (HDACi) in clear cell renal cell cancer (RCC) (Pili et al., 2017), bevacizumab and panobinostat (HDACi) in high-grade glioma (Lee et al., 2015). Besides, the epigenetic drugs could also be combined with the MEK/BRAF inhibitors and PARP inhibitors (Thy et al., 2021). However, the toxicity and tolerability of these drug combinations is the biggest question in clinical trials.

### 4.5 Epigenetic drugs in combination with immunotherapies

The epigenetic regulation was found to overcome drug resistance of the immune-checkpoint blockade (ICB). The combinations of epigenetic drugs and immune-checkpoint inhibitors were demonstrated effective for the treatment of cancers which were refractory or resistant to ICB, both in preclinical and clinical studies. The combinations of HDACi and ICB have received favorable clinical effect in the clinical trials, such as vorinostat and pembrolizumab (anti-PD-1 antibody) for the treatment of ICB-resistant metastatic NSCLC (Rodriguez et al., 2020), entinostat and pembrolizumab for the treatment of microsatellite-stable CRC (Medina Lopez et al., 2022). It is worth mentioning that the combinations of epigenetic drugs and immune-checkpoint inhibitors are usually well tolerated without severe toxic effects, which are superior to the combinations with targeted therapy.

Nonetheless, extended application of epigenetic drugs could induce harmful influence in the antitumor immunity. For instance, the BETi could cause severe depletion of T cells in the tumor environment (Wu et al., 2021). Therefore, the sequential or intermittent dosage regimen was adopted to induce the initiation of the epigenetic regulation and create an anti-cancer microenvironment during the treatment.

## 5 Epigenetic biomarker development

Epigenetic biomarkers are able to provide relevant information for diagnosis, prognosis and therapy optimization in routine clinical treatment and drug discovery. Epigenetic biomarkers may provide a rationale for patient stratification and precision medicine, thus maximizing the chances of treatment success while minimizing unwanted effects. Epigenetic biomarkers can also provide extra advantages, including low patient invasiveness. For example, variations in DNA methylation can be detected in body fluids and liquid biopsies (Liu et al., 2018). The development of accurate measurements of epigenetic alterations of specific targets in patients will greatly guide the clinical application of epigenetic drugs. The DNA repair gene O6-methylguanine-DNA methyltransferase (MGMT) methylation status is the first discovered biomarker in neuro-oncology. The promoter methylation of MGMT in glioblastomas could predict the therapeutic effect of temozolomide (Hegi et al., 2005). It has been shown *in vitro* that azacytidine and decitabine use different human nucleoside transporters (hNTs), and that cytotoxicity is dependent on hNT presence. These observations suggest that hNTs may be useful biomarkers for the efficacy of DNMTis, but clinical data are still not available (Damaraju et al., 2012). Unfortunately, the most extensively studied biomarker for HDACi activity is acetylation levels of the target proteins before and after treatment in peripheral blood or tumor tissue, but no correlation to clinical response has been found. Indeed, hyperacetylation was generally observed in all patients irrespective of response to HDACi (Ellis et al., 2008; Haigentz et al., 2012). The application of patient-stratified epigenetic biomarker, along with predictive models, will take our understanding and use of cancer epigenetics to a new level in the diagnosis, prognosis and treatment of cancer patients.

## 6 Conclusion and future perspectives

**FIGURE 1:**
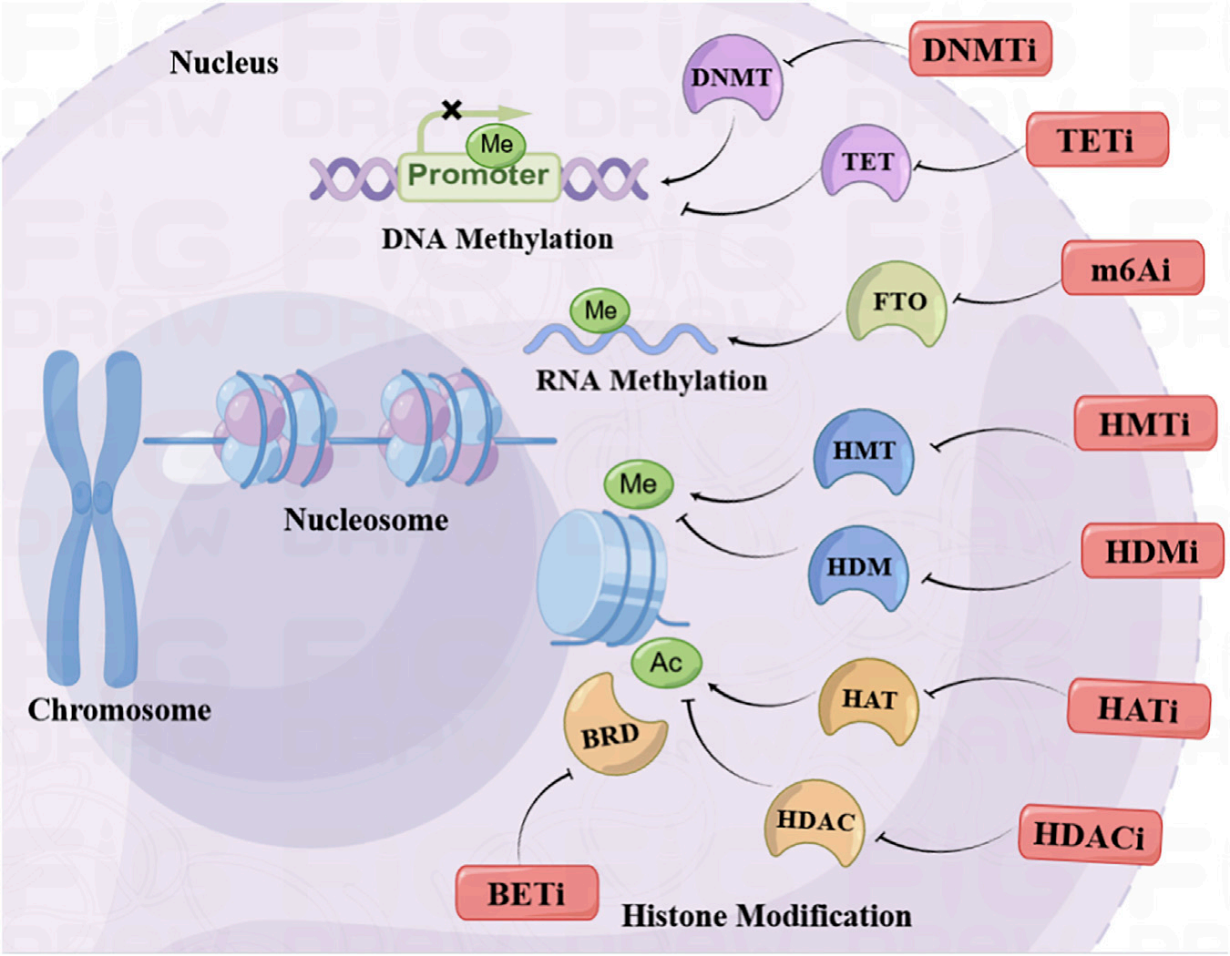
Recent advances in epigenetic anticancer therapeutics.

Although epigenetic drugs have made great progress in cancer drug development, the problems that arise are not to be underestimated. The first and most serious problem is that the low selectivity of epigenetic drugs leads to serious adverse reactions, such as the HDACi. Therefore, the search for epigenetic drugs with better selectivity that can target more elaborate isoform of epigenetic target may be one of the significant development directions in the future. In addition, monotherapy of epigenetic drugs presented favorable efficacy in hematologic cancer rather than in solid tumor. Therefore, the combination of epigenetic drugs and other antitumor therapies in the treatment of insensitive solid tumors and drug-resistant recurrent tumors is in active development. Unfortunately, the occurrence of serious toxic effects is still the main reason that disturbs the application of combined therapy. Hence, the exploration of optimizing the combination regimen and reducing the administered dosage may be promising directions for the extensive application of epigenetic drugs in the future. In summary, this review systematically concluded the recent progress of the epigenetic therapeutics in the treatment of cancers (Table 1) and the combination strategy with other therapies (Figure 1). Epigenetic drugs still have a broad prospect in the treatment of cancers. Optimizing the combination administration regimen to reduce toxic side effects and developing new epigenetic drugs with less toxicity may be two significant directions in the future.
